# Supplementary material for: Are There Effective Interventions to Increase Physical Activity in Children and Young People? An Umbrella Review
Source: Int J Environ Res Public Health. 2020 May 18;17(10):3528. doi: 10.3390/ijerph17103528 (PMC7277151; doi:10.3390/ijerph17103528)

Supplemental Table S1. Qualitative findings of systematic review included.

First Author, Year	Title	Study design*	N°**	Intervetion	Primary outcome	Participants age	Follow-up	Results	Setting***
AJ Atkin, 2011	Interventions to promote physical activity in young people conducted in the hours immediately after school: A systematic review	RCTs, c-RCTs, c-NRT	10	Intevention to promote PA in after school	PA (accellerometer or self-report)	<18 years	/	Of the nine studies meeting inclusion criteria, three reported an increase or smaller decrease in physical activity amongst intervention participants compared with controls and six indicated no change. Evidence suggests that single-behaviour interventions may be most effective during these hours. Overall, findings indicate that interventions to promote physical activity delivered in the after-school setting have been ineffective to date	School
Ávila García, 2016	Programas de intervención para la promoción de hábitos alimentarios y actividad física en escolares españoles de Educación Primaria: revisión sistemática.	/	7	School based interventions to increase PA	BMI, PA	6-12 years	from 2 weeks to 2 years	Of the seven intervention programs analyzed, only three studies achieved statistically significant improvements in body composition. In two studies the rate of BMI significantly improved. Five programs significantly increased daily physical activity	School
Bleich, 2017	Interventions to prevent global childhood overweight and obesity: a systematic review	RCTs, quasi-experimental studies, natural experiments	56	Intervention to promote PA in all setting	BMI, BMI Z score, BMI percentile	Children and adolescent	≥ 12 months for community-based and home-based interventions or ≥ 6 months for school-based and preschool-based interventions	School-based interventions with combined diet and physical activity components and a home element had greatest effectiveness; evidence in support of the effect of preschool-based (n=6), community-based (n=7), and home-based (n=2) interventions was limited by a paucity of studies and heterogeneity in study design	Community setting and miscellaneous
Camacho-Miñano, 2011	Interventions to promote physical activity among young and adolescent girls: A systematic review	RCTs and quasi experimental design	29	School based interventions	Objective or self-reported assessment of PA	Girls aged 5-18 years	from 1 week to three years	Ten of the 21 studies reported a favorable intervention effect upon PA outcomes, seven of which were rated as having a high methodological quality. Five studies rated as having a high methodological quality reported a non-effective result upon PA outcomes, the remaining six ineffective PA interventions were studies rated as low quality	School
Gorga, 2016	School and family-based interventions for promoting a healthy	RCTs and CTs	11	behavioral and/or educational interventions	BMI, weight	6 -18 years	there is one study without	Short-term results are more easily reachable because there is a continuous reinforcement of the message during the	School

	lifestyle among children and adolescents in Italy: a systematic review			aimed at primary prevention of cardiovascular risk factors in children and adolescents			follow-up and other ones from 8 months to 4 years	study period. All RCTs have demonstrated an improvement in BMI. The holistic involvement of children, adolescents, families, and communities achieves a valuable impact on cardiovascular risk prevention among children, suggesting that probably this will be the best approach for future projects.	
Hamel, 2011	Computer- and web-based interventions to increase preadolescent and adolescent physical activity: A systematic review	RCTs or quasi-experimental	14	Computer, web-based interventions at school or at home	BMI, weight, %	8–18 years	from 1 month to 2 years	Most interventions demonstrated statistically significant increases in physical activity or positive health changes related to physical activity, findings were small or short-lived.	Community setting and miscellaneous
Lau, 2011	A Systematic review of information and communication technology-based interventions for promoting physical activity behavior change in children and adolescents	RCTs	9	Interventions employing Internet, email, and/or SMS to increase PA	PA-related cognitive, psychosocial, and behavioral outcomes	6-18 years	2 weeks to 2 years	Evidence supporting the positive effects of ICTs in PA interventions for children and adolescents, especially when used with other delivery approaches (ie, face-to-face). Of the 9 studies, 7 demonstrated positive and significant within-group differences in at least one psychosocial or behavioral PA outcome. In all, 3 studies reported positive and significant between-group differences favoring the ICT group.	Community setting and miscellaneous
Ling, 2015	Interventions to Increase Physical Activity in Children Aged 2-5 Years: A Systematic Review	c-RCTs, RCTSs, quasi-experimental	24	multi-component interventions for pre-school children: structured PA and single-component interventions	objectively measured PA	2-5 years	from 8 weeks to 9 months	The overall intervention effect was less than optimal, the review indicated that theory-driven, multicomponent interventions including a structured physical activity component and targeting both parents and their children may be a promising approach for increasing preschoolers' physical activity	Preschool and childcare center
Martin, 2017	Effect of Active Lessons on Physical Activity, Academic, and Health Outcomes: A Systematic Review	RCTs, no RCT quasi experimental	15	classroom-based physical activity interventions	physical activity, BMI	5–18 years	/	Six studies reporting on physical activity levels were found to have medium-to-large effect sizes. All 4 studies reporting learning outcomes showed positive effects of intervention lessons. Teachers and students were pleased with the programs, and enhanced on-task behavior was identified (n = 3). Positive effects were also reported on students' body mass index levels (n = 3).	School
McIntosh, 2017	Do E-health interventions improve physical activity in young people: a systematic review	RCTs, c.RCT, quasi-experimental	10	E-health interventions	PA levels	until university youngs	3 months to 2 years	Interventions using social cognitive theory showed statistically significant increases in PA. Interventions based on theory of planned beahviour were relatively successful, with only one study not having any significant	School

								improvements in PA. Specific, measurable, achievable, relevant and time-bound (SMART) goal principle was not effective in increasing PA but had positive findings in supplementary outcomes such as goal setting.	
Mehtälä, 2014	A socio-ecological approach to physical activity interventions in childcare: A systematic review	RCTs, quasi-experimental, before/after	23	PA interventions in childcare by applying a socio-ecological approach	PA level proxy-reported or objectively measured	2-6-year-old	/	Fourteen studies found increases in PA levels or reductions in sedentary time, although the changes were modest. The findings of this review indicate that children's PA remained low and did not approach the 180 min/day criteria.	Preschool and childcare center
Pakarinen, 2017	Health game interventions to enhance physical activity self-efficacy of children: a quantitative systematic review	RCTs or quasi-experimental trials	5	Video game (computer, console, mobile)	PA self-efficacy and exercise self-efficacy, BMI	<18 years	/	Four game interventions, employing three active games and one educational game significantly improved PA children self-efficacy. The fifth intervention, employing a game-themed mobile application, showed no intervention effects.	Community setting and miscellaneous
Pang, 2017	Promoting active travel to school: A systematic review (2010-2016)	RCTs	18	Policy to increase active school travel	Physical acitivity level	/	4 weeks to 5 years	ALBD Community Action Model, Preparation and Promotion were reportedly used much more frequently than Policy and Physical projects. Given that change is evident where policy changes are made extended use of the ALBD model is recommended (Preparation, Promotion, Program, Policy and Physical projects).	School
Pucher, 2013	Systematic review: School health promotion interventions targeting physical activity and nutrition can improve academic performance in primary- and middle school children	RCTs, CTs, quasi-experimental	7	School-based health promotion interventions	Physical acitivity level	primary and middle school children	/	Interventions targeting energy balance have small to large effect on physical activity (range of effect size: 0.19-0.75). Effects of different kind of interventions varied in size and across academic domains, as mathe,atics and language scores	School
Schoeppe, 2016	Efficacy of interventions that use apps to improve diet, physical activity and sedentary behaviour: a systematic review	RCTs, CT	4	Intervention using smartphone app to improve diet, physical activity and/or sedentary behaviou	Body weight	8–17 years	10 weeks	One study reported significant between-group improvements in diet, sedentary behaviour and fitness in the app intervention group. Another study reported a significant within-group increase in physical activity, but no significant difference between groups. The remaining two studies reported no significant changes in the behavioural or related health outcomes	Community setting and miscellaneous
Wolfenden, 2017	Strategies for enhancing the implementation of	Randomised or non-randomised	27	educational materials, educational	BMI	children and school staff	/	Among 13 trials reporting dichotomous implementation outcomes-the proportion of schools or school staff implementing	School

	school-based policies or practices targeting risk factors for chronic disease	outreach and educational meetings, school based policies			a targeted policy or practice-the median unadjusted (improvement) effect sizes ranged from 8.5% to 66.6%. Of seven trials reporting the percentage of a practice, program or policy that had been implemented, the median unadjusted effect (improvement), relative to the control ranged from -8% to 43%.

*Study design included in systematic review; **Number of studies of the systematic review; *** Setting according to "Physical Activity Guidelines for Americans Midcourse Report Strategies to Increase Physical Activity Among Youth".

Supplemental Table S2. Quantitative findings of metanalysis included.

First Author, Year	Title	Study design*	N°**	Intervetion/polices	Primary outcome	Participants age	Follow-up	Results	Setting***
Brad Metcalf, 2012	Effectiveness of intervention on physical activity of children: Systematic review and meta-analysis of controlled trials with objectively measured outcomes	RCTs and CT	30	Incorporated a component designed to increase the physical activity of children/adolescents	whole day PA (accelerometers either before or immediately after the end of the intervention period), MPVA	Children-adolescents	At least four weeks in duration	The pooled intervention effect across all studies was small to negligible for total physical activity SMD=0.12, 95% CI (0.04 to 0.20) and small for MPVA: SMD=0.16 95% CI (0.08 to 0.24). Meta-regression indicated that the pooled intervention effect did not differ significantly between any of the subgroups.This review provides strong evidence that physical activity interventions have had only a small effect (approximately 4 minutes more walking or running per day) on children's overall activity levels.	Community setting and miscellaneous
Borde, 2017	Methodological considerations and impact of school-based interventions on objectively measured physical activity in adolescents: a systematic review and meta-analysis	RCTs	13	School based intervetions	Total PA	≥10 years	from five weekdays to eight consecutive days	The pooled effects were small and non-significant for both total physical activity SMD = 0.02 95% CI (-0.13 to 0.18) and for moderate-to-vigorous physical activity SDM = 0.24 95% CI (-0.08 to 0.56). Sample age and accelerometer compliance were significant moderators for total physical activity, with a younger sample and higher compliance associated with larger effects.	School
Brown HE, 2016	Family-based interventions to increase physical activity in children: a systematic review, meta-analysis and realist synthesis	RCTs, C-RCTs, pilot studies	47	Intervention to increase physical activity by actively engaging the family	Self-reported PA.	5–12 years	up to 6 months to 12 months or longer	The meta-analysis (19 studies) demonstrated a significant small effect in favour of the experimental group SMD=0.41 95% CI (0.15 to 0.67). Sensitivity analysis, removing one outlier, reduced this to SMD= 0.29 95% CI (0.14 to 0.45). The qualitative synthesis showed that in the context of family constraints (such as time or scheduling difficulties), a combination of goal-setting and reinforcement intervention strategies was effective in changing physical activity behaviour, through the mechanism of increased motivation.	Family and home
Dellert JC, 2014	Interventions with children and parents to improve physical activity and body mass index: A meta-analysis	RCTs	21	Interventions for parents, children or families, to improve children's physical activity or BMI	PA, BMI	Parents, children or families	From two days to four years	The interventions had no effect on physical activity and BMI: MD=0.04, 95% CI (-2.04 to 2.13). In subgroup analyses, for interventions aimed at parents and children or families, there was a statistically significant effect for physical activity: MD=0.29, 95% CI (0.09 to 0.48), but not BMI: MD=-0.09, 95% CI (-0.37 to 0.19). For interventions with children only, there was a significant effect on BMI: MD=-0.08, 95% CI (-0.16 to -0.01), but no effect on physical activity MD=0.06, 95% CI (-0.08 to 0.20).	Family and home
Dobbins, 2013	School-based physical activity	RCTs	44	School based intervetions	Rates of MPVA during the school	6 to 18 years	Minimum of 12	There was some evidence that school-based physical activity interventions had a positive impact	School

	programs for promoting physical activity and fitness in children and adolescents aged 6 to 18			day, time engaged in MPVA during the school day, and time spent watching television, BMI,		weeks (8 months to four years)	on four of the nine outcome measures. Specifically positive effects were observed for duration of physical activity, television viewing, VO2 max, and blood cholesterol. School-based physical activity interventions led to an improvement in the proportion of children who engaged in MPVA during school hours OR 2.74, 95% CI (2.01, 3.75). Children and adolescents exposed to the intervention spent less time watching television (results range from five to 60 min less per day), and had improved VO2max (results across studies ranged from 1.6 to 3.7 mL/kg per min)	
Finch M, 2016	Effectiveness of centre-based childcare interventions in increasing child physical activity: a systematic review and meta-analysis for policymakers and practitioners	RCTs	16	Interventions with at least one component/strategy aimed at increasing the physical activity level of children	PA (pedometer or accelerometer)	≤ 6 years	From 2 days to one year	Overall interventions implemented in centre based childcare obtained significant results on child physical activity SMD: 0.44 (95% CI: 0.12-0.76). Significant effects were found for interventions that included structured activity (SMD 0.53; 95% CI: 0.12-0.94), delivery by experts (SMD 1.26; 95% CI: 0.20-2.32) and used theory (SMD 0.76; 95% CI: 0.08-1.44).	Preschool and Childcare Center
Lonsdale, 2013	A systematic review and meta-analysis of intervention designed to increase moderate to vigorous physical activity in school physical lesson	Experimental and quasi-experimental studies	14	Interventions to increase the proportion of physical education lesson time spent in MPVA	MVPA spent during school lessons (accelerometer or systematic direct observation by an independent rater)	6 - 18 years	/	All of the included studies reported a higher proportion of active learning time in the intervention group compared with the control group; most of these differences were reported as being statistically significant. The meta-analysis revealed an absolute difference of 10.37% CI (6.33 to 14.41) of lesson time spent in MPVA in favour of the intervention over controls. The 10.37% difference related to 24% more active learning time in the intervention groups than in the control groups SMD= 0.62, 95% CI (0.39 to 0.84).	School
Mei, 2016	The impact of long-term school-based physical activity interventions on body mass index of primary school children - a meta-analysis of randomized controlled trials	RCTs	18	School-based PA interventions	BMI	6 -10 years	12 to 72 months	The BMI increment was 2.23 kg/m2 less in the intervention groups. Decrement -2.23 CI (-2.92 to -1.55).	School
Oosterhoff, 2016	The effects of school-based lifestyle interventions on body mass index	RCTs	91	School-based lifestyle interventions	BMI	4-12 years	/	The pooled effects were OR=-0.072 95% CI (-0.106; -0.038) for BMI, OR=-0.183 95% CI (-0.288; -0.078) for systolic blood pressure and -0.071 95% CI (-0.185; 0.044) for diastolic blood pressure. In multivariate analyses, the pooled effects of	School

	and blood pressure: a multivariate multilevel meta-analysis of randomized controlled trials							interventions were -0.054 95% CI (-0.131; 0.022) for BMI, OR=-0.182 95% CI (-0.266; -0.098) for systolic blood pressure and -0.144 95% CI (-0.230; -0.057) for diastolic blood pressure. Parental involvement accentuated the beneficial effects of interventions.	
Owen, 2017	The effectiveness of school-based physical activity interventions for adolescent girls: A systematic review and meta-analysis	RCTs, c-RCTs, quasi experimental	20	school based intervetions	Self-report frequency of weekly leisure-time PA	11- 18 years, adolescent girls	from 1 month to 36 months	There was a significant small positive treatment effect for school-based PA interventions for adolescent girls (k=17, g=0.37, p<0.05). After an outlier was removed (residual z=7.61) the average treatment effect was significantly reduced, indicating a very small positive effect 0.07 95% CI (0.00, 0.14). Subgroup analysis revealed very small significant effects for multi-component interventions (k=7, g=0.09, p<0.05), interventions underpinned by theory (k=12, g=0.07, p<0.05), and studies with a higher risk of bias (k=13, g=0.09, p<0.05). Intervention effects were very small which indicates that changing PA behaviors in adolescent girls through school-based interventions is challenging.	School
Plotnikoff, 2015	Effectiveness of interventions targeting physical activity, nutrition and healthy weight for university and college students: a systematic review and meta-analysis	/	41	University interventions	PA, MVPA, VA	18-25 years	all lengths	total physical activity: SMD −0.11, 95% CI: (−0.30 to 0.08) vigorous physical activity: SMD 0.28 95% CI (−0.08 to 0.63), moderate physical activity: SMD 0.18, 95% CI (0.06 to 0.30)' four of twelve studies which reported weight outcomes reported significant improvements.	School
Russ, 2015	Systematic Review and Meta-Analysis of Multi-Component Interventions Through Schools to Increase Physical Activity	RCTs and noRCTs	14	Programs ≥ 2 CSPAP components (comprehensive school physical activity), with at least 1 targeting school-based PA during school hours (physical education (PE), PA during the school day (PADS), PA before/after school (PABAS), staff wellness (SW), and family/community engagement (FCE))	Total daily PA, MVPA, sedentary activity levels	5-18 years	Median 360 days	Daily PA was minimal, with a pooled effect size of g = 0.11, 95% CI (0.03 to 0.19). Comparable effects were observed for studies that reported daily physical activity for boys g = 0.09, 95% CI (-0.10 to 0.28) and girls g = 0.11, 95% CI (-0.02 to 0.23), separately, and in studies that reported boys and girls combined g = 0.12, 95% CI (0.05 to 0.19). Evaluation of the inclusion of each specific CSPAP component found that studies that included PADSD (0.19 vs. 0.07), PABAS (0.29 vs. 0.10), and SW (0.21 vs. 0.09) were associated with larger effect sizes than studies that did not include these components. The only CSPAP component associated with a smaller effect size was QPE (0.10 vs. 0.16).	School

Waters, 2011	Interventions for preventing obesity in children	RCTs and CT	55	School based intervetions	BMI	<18 years	twelve weeks or more	Children in the intervention group had for BMI a SMD=-0.15 95% CI (-0.21, -0.09). Intervention effects by age subgroups were -0.26 95% CI (-0.53, 0.00) (0-5 years), -0.15 95% CI (-0.23, -0.08) (6-12 years), and -0.09 95% CI (-0.20, 0.03) (13-18 years).	School
Watson A, 2017	Effect of classroom-based physical activity interventions on academic and physical activity outcomes: a systematic review and meta-analysis	RCTs, C-RCTs, quasi experimental,	39	Classroom-based physical activity	PA	5–12 years	Until 3 years	The meta-analyses showed classroom-based physical activity had a positive effect on improving on-task and reducing off-task classroom behaviour OR=0.60, 95% CI (0.20 to 1.00), and led to improvements in academic achievement SMD = 1.03, 95% CI (0.22,1.84). However, no effect was found for cognitive functions SMD = 0.33, 95% CI (-0.11,0.77) or physical activity OR: 0.40, 95% CI (-0.15 to 0.95).	School
Williams AJ, 2013	Systematic review and meta-analysis of the association between childhood overweight and obesity and primary school diet and physical activity policies	Observational studies and RCTs	21	Diet or physical activity related school policies either alone or as part of intervention programmes	BMI	4 to 11 years	≥6 months	National School Lunch Program BMI, OR: 0.04 95% CI (-0.193 to 0.27), School breakfast program, BMI, OR: −0.080, 95% CI (−0.143 to −0.017), other diet related policies BMI OR: −0.021, 95% CI (−0.066 to 0.023). The pooled effects of the physical activity, and other diet related policies on BMI-SDS were non-significant.	School

*Study design included in metanalysis; **Number of studies; PA: physical activity; BMI: body mass index.

Supplemental Table S3. Quality assessment by AMSTAR2.

	Values* of the items of AMSTAR 2**																Final rate***
**Author, year**	**1**	**2**	**3**	**4**	**5**	**6**	**7**	**8**	**9**	**10**	**11**	**12**	**13**	**14**	**15**	**16**	
Atkin, 2011	/	/	/	2	/	/	/	1	2	/	NA	NA	/	/	NA	/	Critically low
Ávila García, 2016	/	/	/	/	1	/	/	/	/	/	NA	NA	/	/	NA	/	Critically low
Bleich, 2017	1	/	1	2	1	1	/	1	1	/	NA	NA	/	1	NA	1	Critically low
Borde, 2017	1	/	/	2	1	1	/	2	1	/	1	1	1	1	/	1	Critically low
Metcalf, 2012	1	/	1	2	1	1	/	1	1	/	NA	NA	/	/	NA	1	Critically low
Brown HE, 2016	1	1	/	2	1	1	/	1	/	/	1	1	1	1	1	1	Critically low
Camacho-Miñano, 2011	/	/	/	/	/	/	/	/	2	/	NA	NA	1	1	NA	1	Critically low
Dellert JC, 2014	1	/	1	/	1	/	/	1	/	/	1	/	/	1	1	1	Critically low
Dobbins, 2013	1	1	1	1	1	1	1	1	1	/	NA	NA	1	1	NA	0	High
Finch M, 2016	1	1	1	/	1	1	/	2	1	/	1	1	1	1	1	1	Critically low
Gorga, 2016	/	/	1	/	1	1	/	1	/	/	NA	NA	/	/	NA	/	Critically low
Hamel, 2011	1	/	1	/	1	1	/	2	/	/	NA	NA	/	/	NA	1	Critically low
Hong Mei, 2016	/	2	1	/	/	/	/	2	1	/	1	/	/	1	1	1	Critically low
Lau, 2011	1	/	/	2	1	1	/	1	/	/	NA	NA	1	1	NA	1	Critically low
Ling, 2015	1	/	1	2	1	1	/	1	2	/	NA	NA	1	1	NA	/	Critically low
Lonsdale C1, 2013	1	/	/	/	1	1	/	1	2	/	1	1	1	1	1	1	Critically low
Martin, 2017	1	/	1	2	1	/	/	1	1	/	NA	NA	1	/	NA	/	Critically low
McIntosh, 2017	1	1	1	2	1	1	/	1	/	/	NA	NA	1	/	NA	1	Critically low
Mehtälä, 2014	1	/	1	2	1	1	/	1	1	/	NA	NA	/	/	NA	1	Critically low
Oosterhoff, 2016	1	/	/	/	1	1	1	2	1	/	1	1	/	1	1	1	Low
Owen, 2017	/	1	/	/	/	1	/	2	1	/	1	1	1	1	1	1	Low
Pakarinen,	1	/	/	1	1	1	/	1	1	/	NA	NA	1	1	NA	1	Critically low
Pang, 2017	1	/	/	2	/	1	/	1	1	/	NA	NA	1	1	NA	1	Critically low
Plotnikoff, 2015	/	/	/	2	1	1	/	/	1	/	1	1	/	1	/	1	Critically low
Pucher,2013	1	/	1	1	1	/	/	2	/	/	NA	NA	/	1	NA	/	Critically low
Russ, 2015	/	/	/	/	1	1	/	/	1	/	1	/	/	/	/	/	Critically low
Schoeppe, 2016	/	/	1	2	1	1	2	2	1	/	NA	NA	1	/	NA	1	Low
Waters, 2011	1	1	1	1	1	1	1	1	1	/	1	1	1	1	1	1	High
Watson, 2017	1	1	/	/	1	/	/	2	2	/	1	1	/	/	/	1	Critically low
Williams, 2013	/	2	1	/	1	1	/	/	2	/	1	1	1	1	/	1	Critically low
Wolfenden, 2017	1	1	1	1	1	1	1	1	1	1	NA	NA	1	1	NA	1	High

Quantitative value of each item*

“NA” non applicable

“/” no

“1” partial yes

”2” yes

Items of AMSTAR 2**

1 Did the research questions and inclusion criteria for the review include the components of PICO?

2 Did the report of the review contain an explicit statement that the review methods were established prior to the conduct of the review and did the report justify any significant deviations from the protocol?

3 Did the review authors explain their selection of the study designs for inclusion in the review?

4 Did the review authors use a comprehensive literature search strategy?
5 Did the review authors perform study selection in duplicate?
6 Did the review authors perform data extraction in duplicate?
7 Did the review authors provide a list of excluded studies and justify the exclusions?
8 Did the review authors describe the included studies in adequate detail?
9 Did the review authors use a satisfactory technique for assessing the risk of bias (RoB) in individual studies that were included in the review?
10 Did the review authors report on the sources of funding for the studies included in the review?
11 If meta-analysis was performed did the review authors use appropriate methods for statistical combination of results?
12 If meta-analysis was performed, did the review authors assess the potential impact of RoB in individual studies on the results of the meta-analysis or other evidence synthesis?
13 Did the review authors account for RoB in individual studies when interpreting/ discussing the results of the review?
14 Did the review authors provide a satisfactory explanation for, and discussion of, any heterogeneity observed in the results of the review?
15 If they performed quantitative synthesis did the review authors carry out an adequate investigation of publication bias (small study bias) and discuss its likely impact on the results of the review?
16 Did the review authors report any potential sources of conflict of interest, including any funding they received for conducting the review?

Final rate evaluation***

High

No or one non-critical weakness: the systematic review provides an accurate and comprehensive summary of the results of the available studies that address the question of interest

Moderate

More than one non-critical weakness*: the systematic review has more than one weakness but no critical flaws. It may provide an accurate summary of the results of the available studies that were included in the review

Low

One critical flaw with or without non-critical weaknesses: the review has a critical flaw and may not provide an accurate and comprehensive summary of the available studies that address the question of interest

Critically low

More than one critical flaw with or without non-critical weaknesses: the review has more than one critical flaw and should not be relied on to provide an accurate and comprehensive summary of the available studies.

Supplemental Figure S1. PRISMA 2009 Flow Diagram

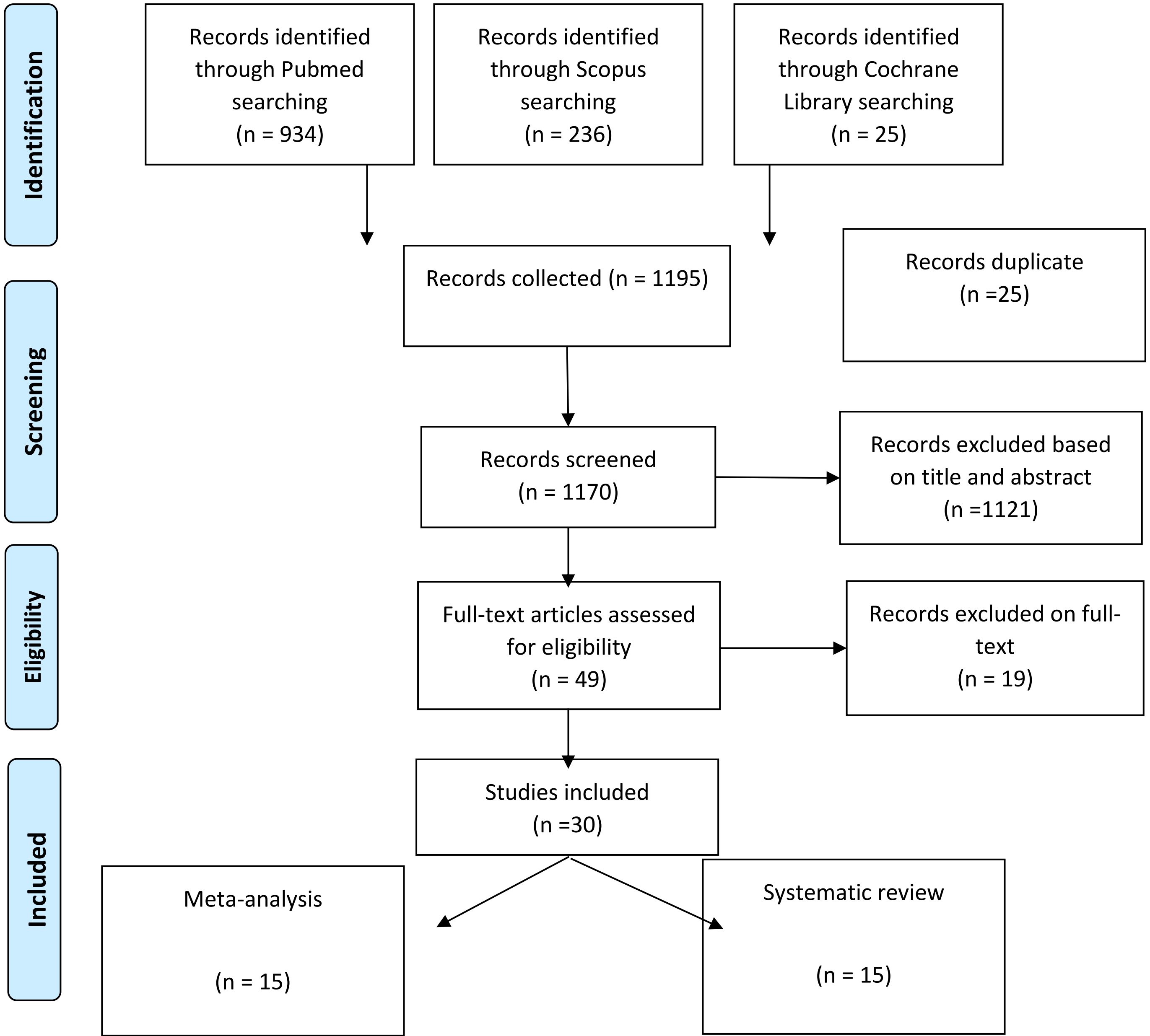

Supplement: Supplementary file 1 [file ijerph-17-03528-s001.pdf]
